# Urinary Cotinine Concentration and Self-Reported Smoking Status in 1075 Subjects Living in Central Italy

**DOI:** 10.3390/ijerph15040804

**Published:** 2018-04-19

**Authors:** Enrico Paci, Daniela Pigini, Lisa Bauleo, Carla Ancona, Francesco Forastiere, Giovanna Tranfo

**Affiliations:** 1 INAIL, Department of Occupational and Environmental Medicine, Epidemiology and Hygiene, Via di Fontana Candida 1, Monteporzio Catone, 00078 Rome, Italy; e.paci@inail.it (E.P.); d.pigini@inail.it (D.P.); 2 Lazio Regional Health Service, Department of Epidemiology, Via Cristoforo Colombo 112, 00147 Rome, Italy; l.bauleo@deplazio.it (L.B.); c.ancona@deplazio.it (C.A.); fran.forastiere@gmail.com (F.F.)

**Keywords:** biomarkers, urinary cotinine, environmental tobacco smoke, smoking, cutoff, HPLC-MS/MS

## Abstract

*Background:* Urinary cotinine, a metabolite of nicotine, is a marker of tobacco smoke exposure. A cutoff value for cotinine concentration can be set to distinguish smokers from non-smokers, independently from self-declared status. *Method:* Cotinine was determined by isotopic dilution High Performance Liquid Chromatography coupled to tandem mass spectrometry (HPLC-MS/MS) between 2013 and 2014 on urine samples of a population of 1075 subjects. *Results*: 296 subjects have a cotinine level higher than 100 μg/g of creatinine (cutoff), with a median cotinine concentration of 1504.70 μg/g of creatinine. The mean is 27.5% of smokers and 60.5% in this group are females. The median value for non-smokers is 5.6 μg/g of creatinine. Two hundred and seventy-five subjects declared to be smokers in the questionnaire, but 6 (2.2%) present urinary cotinine levels lower than cutoff; 800 subjects declared to be non-smokers, but 26 of them presented urinary cotinine levels that were higher than the cutoff (3.3%). *Conclusion:* Using the cutoff of 100 μg/g, the misclassification of smokers resulted to be 2.2%, indicating that the selected value is suitable for studying the human exposures to environmental and occupational pollutants, including those produced by smoking.

## 1. Introduction

Cigarette smoke is the cause of 90% of all lung cancer death. In fact, tobacco smoke contains, among thousands of chemicals, 44 compounds that were classified as human carcinogens by the International Agency for Research on cancer (IARC). Environmental tobacco smoke, also known as passive smoking, is partly produced by the burning end of the cigarette (or pipe or cigar) and partly exhaled by the smokers [1]. According to the World Health Organization, tobacco causes almost 6 million deaths each year, 10% of which can be attributed to passive smoking [2]; exposure to both active and passive smoking can also cause cardiovascular and respiratory diseases, and its effects are more severe in children and newborns [3].

The assessment of the smoking status of a subject is needed for documenting the extent of the exposure to tobacco smoke, both for monitoring the progress of tobacco control programs and because smoking is a major confounding factor in the assessment of exposure to important occupational and environmental pollutants, like benzene, formaldehyde, polycyclic aromatic hydrocarbons, and some heavy metals. For example, the median urinary value of S-phenyl-mercapturic acid (SPMA), the most specific human metabolite of benzene, is about ten times higher in smokers than in non-smokers, and its concentration is linearly correlated to that of urinary cotinine in smokers [4]. A strong influence of smoking on the urinary biomarkers of the polycyclic aromatic Hydrocarbons 1 and 2 naphthalene and pyrene, (1-OH-Naphthalene, 2-OH-Naphthalene, and 1-OH-Pyrene) was observed in a biomonitoring study on 200 volunteers conducted using HPLC-MS/MS [5].

Self-reported smoking status may not always represent a subject’s true smoking status, and for this reason, the level of the urine cotinine is used as a biomarker of tobacco smoke exposure. In humans, cotinine is one of the most important metabolites of nicotine, a major component of tobacco smoke, metabolized in the liver by the enzyme cytochrome P450 2A6 (CYP2A6). Cotinine can be excreted in the urine as an N-glucuronide conjugate and it accounts for about 10–15% of the sum of the nicotine excreted unchanged plus the other metabolites; it could also be measured in blood, urine, saliva, hair, or nails. The dosage of cotinine concentration in the body fluids indicates a recent exposure to tobacco smoke, while a long-term exposure information can be obtained from the dosage of nicotine concentration in nails and hair [6].

It is possible to set an optimal cut-off value for the urinary cotinine concentration to distinguish smokers from non-smokers and to validate their self-reported smoking status: the urinary cotinine concentration depends on the smokers’ behavior or on the extent of exposure to secondhand smoke. In passive smokers, the cotinine concentration is lower than 50 µg/L of urine while in smokers, the cotinine concentration is generally more than 100 µg/L [7].

We conducted a human biomonitoring study measuring urinary levels of cotinine by HPLC-MS/MS in a population of 1075 subjects in order to assess their exposure to active and/or passive smoking and to understand the reason for possible misclassifications using a cutoff value of 100 µg/g of creatinine for active smokers.

## 2. Methods

### 2.1. Study Population

The study is a part of the larger “*ABC biomonitoring study*” carried out from May 2013 and December 2014 on a population of 2000 subjects randomly selected from about 130,000 inhabitants of Civitavecchia (Italy); the study protocol was approved by the local ethics committee [8] of AUSL RM/E on 16 April 2013, and registered as n. 14/13.

One thousand and seventy-five subjects out of two thousand agreed to participate in the study. They released a written informed consent and filled in a questionnaire for collecting information on age; lifestyle; food habits; cigarette, cigar, or tobacco smoking; the starting age for smoking; the end age for ex-smokers; electronic cigarette smoking; passive smoking; drug use; working activities; hobbies; and the use of chemical products. Some of this information was collected for the purpose of studying the exposure to different chemical pollutants and were not used in the present study.

### 2.2. Urine Sample Collection

The first urine of the morning was collected by fasting subjects on the day of the medical visit, in empty plastic sterile containers; 30 mL of each sample was transferred into 50 mL Teflon tubes, identified with the subject code, frozen at −20 °C and later transported to the laboratory where they were stored at −20 °C until analysis.

### 2.3. Analytical Methods

The concentration of cotinine was determined by isotopic dilution HPLC-MS/MS following an analytical method previously validated in our laboratories, with the objective to measure simultaneously, on a single urine sample, both the benzene metabolite SPMA and the cotinine [4]. The urine samples were subjected to SPE purification on Sep-pack C18 cartridges. The cartridge was preconditioned, washed, and eluted in order to obtain a first acidic fraction containing SPMA; afterward, the cartridge was washed again, and the first washing fraction containing the cotinine and its internal standard at pH = 8, was loaded onto the cartridge and eluted with methanol. After filtration on Anotop syringe filters, the eluate was injected into the HPLC/MS-MS system and equipped with an HPLC Series 200 LC (PerkinElmer, Norwalk, CT, USA) that was coupled with a tandem mass spectrometer with Turbo ion spray source (TIS) (API 4000, AB/Sciex, Concorde, ON, Canada). Chromatographic separation was performed on a Sinergy Fusion C18 analytical column (150 × 4.6 mm, 4 μm) using a gradient of acetonitrile and acetic acid at 1.0% in water *v*/*v*. The ionic transitions monitored (precursor → product) are, in the negative mode, 177.3 → 80.10 for cotinine and 180.3 → 80.10 for cotinine −d3; the 1.5 version of Analyst*^®^* software (AB/Sciex, Concorde, ON, Canada) was used for instrument control. The final concentration of cotinine is expressed in µg/g of creatinine in order to normalize the results for the different dilution grades of the urine samples. Urinary creatinine was determined by the method of Jaffè with alkaline picrate test and UV/Vis detection at 490 nm [9].

### 2.4. Statistical Analysis

A descriptive statistic was carried out using the Analysis ToolPak, a Microsoft Office Excel (Microsoft, Washington, WA, USA) add-in program. Prior to performing any other statistical analysis, the normality of the distribution of the concentrations of the analyte was evaluated. The cotinine concentration is presented as mean with its standard deviation (SD), median, and 95th percentile.

The frequency of the cotinine levels of all subjects was also represented as a histogram.

In order to compare the results of the classifications made using the questionnaire and using the cotinine level, the Cohen’s kappa coefficient, a statistical measure of inter-annotator agreement for categorical items, was applied.

## 3. Results

The characteristics of the population in terms of gender, age, and occupation is reported in Table 1. Subjects have been classified both according to the self-reported smoking status in the questionnaire (columns 2 and 3) and according to the urinary concentration of cotinine (columns 4 and 5). We defined smokers as subjects having urinary cotinine of ≥100 µg/g.

The descriptive statistics of the cotinine concentration levels of the subjects have been reported in Table 2, stratified by the smoking status assessed with both methods (questionnaire and cutoff).

According to the questionnaire there are 275 smokers, but 6 of these present urinary cotinine levels lower than the cutoff, giving a 2.2% misclassification rating; 800 subjects declared to be non-smokers, but 26 of them present urinary cotinine levels higher than the cutoff (3.3%).

According to the cutoff criterion, the number of smokers is 296, 26 of which had declared to be non-smokers (8.8%). There were 779 non-smokers, 6 of which had declared to be smokers (0.8%). Among these 779 subjects, 96 had declared to be exposed to passive smoking.

The rate of agreement between the classification as a smoker/non-smoker obtained with the measurement of urinary cotinine and self-assessed in the questionnaire was determined by the Cohen’s kappa coefficient that resulted to be 0.8817, providing an agreement of 95.44%.

**Figure 1:**
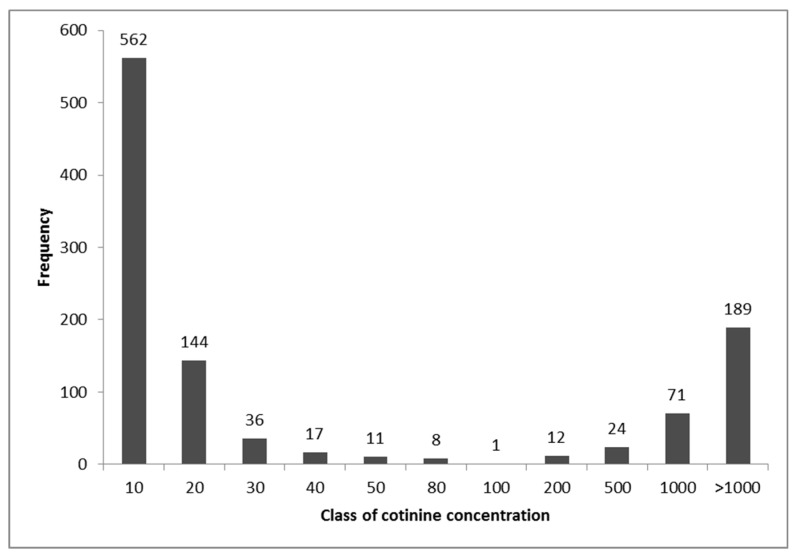
The histogram of the frequency for urinary cotinine levels of all subjects.

The frequency of the cotinine levels for all subjects was plotted in a histogram (Figure 1).

A more comprehensive analysis of the data considering gender, age, and job was performed, and a significant difference (*t*-test, *p* < 0.005) was found between the urinary concentration of cotinine of males and females in the group having urinary cotinine levels lower than the cutoff. No other significant differences were found.

## 4. Discussion

The data presented indicate that in a group of 1075 subjects living in central Italy, there are 27.5% of smokers, 60.5% of which are females.

In the first group of Table 2 (self-reported smokers), there are 6 subjects that were misclassified (low cotinine levels) and they are exactly the same subjects that were “misclassified” according to the cutoff criterion. These subjects have a mean cotinine level of 24.3 µg/g creatinine and they declared to smoke 1–6 cigarettes per day (mean of 2.5). The reason for this misclassification, even if to a small extent, is clearly the few cigarettes smoked and maybe also to their low nicotine content.

In the second group (self-reported non-smokers), 26 subjects were misclassified, 18 of which are ex-smokers and they are the same persons misclassified in the third group (cutoff smokers); it is possible that ex-smokers are exposed to passive smoking by their friends or relatives who are still smoking and that sometimes, they “fall into temptation”. The remaining 8 subjects never smoked, according to the questionnaire, but 3 of them declared to be exposed to passive smoking.

In the third group (cutoff smokers), 8 subjects (not the same 8 of the first group) claimed to have stopped smoking since more than one year, and 3 of them currently smoke electronic cigarettes.

The misclassification of non-smokers with high cotinine level can be therefore be explained by exposure to passive smoking (even if not declared), use of electronic cigarettes that can contain nicotine, metabolic differences, or analytical factors.

There is an individual variability in the percentage of nicotine converted to cotinine (between 50% and 90%) because different subjects metabolize the nicotine at different rates, from 20 to 75 mL/min. This leads to a variability in the quantitative relationship between nicotine intake and cotinine excretion [10].

Besides, as cotinine is excreted in the urine also as an *n*-glucuronide conjugate, the sample storage time and temperature can influence the spontaneous hydrolysis of the conjugate, increasing the cotinine concentration of the sample, independently from the nicotine intake [11].

On the other side, 96 subjects having a cotinine concentration lower than the cutoff have declared to be exposed to passive smoking. This means that the risk perception can be greater than the risk itself and also that our cutoff is diagnostic for daily active smokers, while passive smokers are classified as non-smokers.

The agreement between the questionnaire and the cotinine classification is good, confirming the willingness of the participants to cooperate with this study.

In order to confirm the goodness of the cutoff value of 100 µg/g creatinine, we collected in Table 3, a summary of the studies published from 2012 to 2017 reporting urinary cotinine cutoff values in population studies. The values are reported in ng/mL go from a minimum of 27 (for occasional smokers) to a maximum of 200.

The value of 100 μg/g of creatinine has been determined in a previous study where the correlation between cotinine and benzene metabolite SPMA was explored [4]: the comparison with the cutoff values reported in Table 3 is not immediate, as we expressed it normalized for the urinary creatinine content. However, if we consider that the average content of creatinine of a urine sample is 1 g/L, the corresponding value would be 100 ng/mL. As a general consideration, the study findings reported in Table 3 suggest that a geographic factor should also be considered in the cutoff determination, as this value is linked to the median urinary cotinine concentration of smokers that can be significantly different in studies carried out in different countries even if during the same period of 5 years.

The cutoff value should be set on the basis of the objective of the study; when the objective is the assessment of the daily active smoker status, a higher value can be used, like in the large population studies as the KHANES studies [12,13,16] and in the studies where the main objective is the exposure assessment to chemicals for which smoking is a confounding factor, as in our previous paper [4].

When the objective of the study is the cessation of smoking, or if it is related to health issues, occasional smokers and passive smokers should also be identified. Therefore, a lower cutoff value should be used, like in the studies that examined pregnant women, mothers, and children [14,15,17]. Our results suggest that the 95th percentile of urinary cotinine of the non-smokers group could be used, that is, 24 μg/g of creatinine for males and 35 μg/g of creatinine for females.

The recent study of Campo et al. [18] carried out in Italy found a good cutoff at 30 ng/mL, close to the value of 95th percentile of the urinary cotinine concentration of the “true” non-smokers group of the present study, equal to 28 μg/g of creatinine. However, the median urinary cotinine levels in the smokers group of Campo et al. was 883 ng/mL, a lower value than the median level found in the present study.

The distribution of the results of the present study reported in the histogram shows that the number of subjects having a urinary cotinine concentration between 50 and 200 μg/g of creatinine is 32, only 3% of the total, and therefore, using a cutoff value in this range the misclassification will be negligible.

## 5. Conclusions

In conclusion, we found a good agreement between the questionnaire and the cotinine classification, as in this study, the participants were willing to cooperate. However, in controversial situations lacking the possibility to administer a questionnaire, a method for a non-subjective and quantitative assessment of the smoke exposure of the subjects is indispensable. Subjects who are declared to be non-smokers, but classified as smokers according to the cutoff, are apparently not aware of the influence of passive smoking, nicotine-containing electronic cigarettes, and occasional smoking on this parameter. This should be also considered in the campaign for smoking cessation in the workplaces and in other initiatives for the general population.

In biomonitoring studies for the assessment of exposure to occupational and environmental pollutants like benzene, formaldehyde, polycyclic aromatic hydrocarbons, and some heavy metals, quantitative information is also of great help in the interpretation of results.

Using a cutoff of 100 μg/g of creatinine, only very light smokers were classified as non-smokers, resulting in a 2.2% misclassification. This cutoff value is suitable for general population studies in which daily active smokers have to be identified. When the objective of the study is the cessation of smoking, or when it is related to health issues, very light smokers and passive smokers should also be identified and a lower cutoff should be set.

However, due to the small number of subjects having cotinine levels between 50 and 200 μg/g of creatinine, the choice of the cutoff in this range is not critical, in accordance with the other studies examined.

## Figures and Tables

**Table 1:** The characteristics of the studied subjects and the smoking status.

	Self-Reported Smoking Status		Urinary Cotinine Concentration µg/g Creatinine	
All Subjects	Smokers	Non-Smokers	≥100	<100
1075	275	800	296	779
Sex, *n* (%)				
Male	106 (38.6)	354 (44.3)	117 (39.5)	343 (44.0)
Female	169 (61.4)	446 (55.7)	179 (60.5)	436 (56.0)
Age, *n* (%)				
35–44	72 (26.2)	161 (20.1)	78 (26.4)	155 (19.9)
45–54	88 (32.0)	246 (30.8)	93 (31.4)	241(30.9)
55–64	87 (31.6)	236 (29.5)	90 (30.4)	233 (29.9)
≥65	28 (10.2)	157 (19.6)	35 (11.8)	150 (19.3)
Jobs, *n* (%)				
Employed	110 (40.0)	309 (38.6)	124 (41.9)	295 (37.9)
Autonomous workers	36 (13.1)	72 (9.0)	40 (13.5)	74 (9.5)
Unemployed	32 (11.6)	72 (9.0)	31 (10.5)	71(9.1)
Housewives	50 (18.2)	161 (20.1)	58 (19.6)	159 (20.4)
Retired	47 (17.1)	186 (23.3)	43 (14.5)	180 (23.1)

**Table 2:** The urine Cotinine concentration and smoking status classification.

	Self-Reported Smokers	Self-Reported Non-Smokers	Cutoff Smokers (Cotinine ≥ 100)	Cutoff Non-Smokers (Cotinine < 100)
Total *n*.	275	800	296	779
Mean cotinine (SD) µg/g creatinine	2732.50 (3451.20)	35.07 (195.85)	2608.30 (3361.40)	9.18 (10.38)
Median	1589.3	5.88	1504.70	5.60
95th percentile	9317.00	46.78	9219.00	27.84
Misclassification *	6/275	26/800	26/296	6/779
Mean cotinine (SD) µg/g creat.	24.31 (22.54)	805.30 (764.50)	871.3 (774.76)	24.31 (22.54)
Median	19.15	630.50	698.50	19.15
95th percentile	54.75	2323.40	2323.40	54.75
Reason	few/light cigarettes	18 ex-smokers 3 passive smokers 5 possible analytical factors	26 ex-smokers 3 smoke e-cig	few/light cigarettes

* Self-reported smoking status does not agree with the classification based on the cutoff of 100 µg/g creatinine for smokers.

**Table 3:** The urine cotinine cutoff values (2012–2017).

Author	Year	Study/Population Characteristics		CutoffValue (ng/mL)	Ref.
		Description	Subjects		
Sungmo Jung et al.	2012	National Health and Nutrition Examination Survey (KNHANES) investigated from 2007 to 2010 (Korea)	33,829	Males: 95.6 Females: 96.8	[12]
Kim and Jung.	2013	Participants in Korea National Health and Nutrition Examination Survey (KNHANES) for 2008–2010, Korea	11,629	164	[13]
Aurrekoetxea et al.	2013	Pregnant women (INMA Project) interviewed between 2004 and 2008	2263	Smokers: 82 Occasional smokers: 27	[14]
Stragierowicz et al.	2013	Pregnant women (Polish Mother and Child Cohort Study)	69	42.3	[15]
Kim et al.	2014	Korean National Health and Nutrition Examination Surveys IV and V (2008–2010)	4584	50–200	[16]
Lupsa et al.	2015	DEMOCOPHES Study of children and their mothers (120 in each of the three countries), Poland (PL), Portugal (PT) and Romania (RO)	360	All mothers PL 4.4/PT 7.9/ RO 254.2	[17]
Campo et al.	2016	The behavioral and sociodemographic factors (urinary cotinine levels in active smokers and in environmental tobacco smoke)	495	30	[18]
Tranfo et al.	2017	Study performed in the 2 years period 2013–2014.	446	Smoker: ≥100 μg/g of creatinine.	[4]
